# The dose of HBV genome contained plasmid has a great impact on HBV persistence in hydrodynamic injection mouse model

**DOI:** 10.1186/s12985-017-0874-6

**Published:** 2017-10-25

**Authors:** Lei Li, Sheng Li, Yun Zhou, Lu Yang, Di Zhou, Yan Yang, Mengji Lu, Dongliang Yang, Jingjiao Song

**Affiliations:** 1 Experimental Medicine Center, Tongji Hospital of Tongji Medical College, Huazhong University of Science and Technology, Wuhan, 430030 China; 2 Department of Infectious Diseases, Union Hospital of Tongji Medical College, Huazhong University of Science and Technology, Wuhan, China; 3 Department of Infectious Disease, Anhui Provincial Hospital, Anhui Medical University, Hefei, China; 4 Institute of Virology, University Hospital of Essen, University Duisburg-Essen, Essen, Germany

**Keywords:** HBV mouse model, Hydrodynamic injection, The doses of HBV plasmid, HBV persistence

## Abstract

**Background:**

Hydrodynamic injection (HI) of hepatitis B virus (HBV) mouse model is an useful tool for HBV related research in vivo. However, only 40% of C57/BL6 mice injected with 10 μg HBV genome contained plasmid (pAAV-HBV1.2), serum HBsAg more than 6 months and none of the BALB/c mice injected with 10 μg pAAV-HBV1.2 plasmid DNA, serum HBsAg positive more than 4 weeks in the previous study.

**Methods:**

In this study, C57/BL6 and BALB/c mice were hydrodynamic injected with different doses of pAAV-HBV1.2 plasmid DNA. HBV related serum markers were detected by ELISA. ALT levels in the serum were measured using full automated biochemistry analyzer. HBcAg positive cells in the liver were detected by immunohistochemical staining. The mRNA levels of IRF3, ISGs including ISG15, OAS, PKR and immune factors including IFNγ, TNFα, TGFβ, IL-6, IL-10, PDL1 in liver of the mice were quantified by qRT-PCR.

**Results:**

The results showed that the mice injected with 100 μg high-concentration or 1 μg low-concentration of pAAV-HBV1.2 plasmid DNA did not excert dominant influence on HBV persistence. In contrast, injection of 5 μg intermediate-dose of pAAV-HBV1.2 plasmid DNA led to significant prolonged HBsAg expression and HBV persistence in both C57/BL6 (80% of the mice with HBsAg positive more than 6 months) and BALB/c (60% of the mice with HBsAg positive more than 3 months) mice. IFNγ was significant up-regulated in liver of the mice injected with 1 μg or 100 μg pAAV-HBV1.2 plasmid DNA. TNFα was up-regulated significantly in liver of the mice injected with 100 μg pAAV-HBV1.2 plasmid DNA. Moreover, PDL1 was significant up-regulated in liver of the mice injected with 5 μg pAAV-HBV1.2 plasmid DNA.

**Conclusion:**

In this paper we demonstrated that, in the HBV HI mouse model, the concentration of injected pAAV-HBV1.2 plasmid DNA contributes to the diverse kinetics of HBsAg and HBeAg in the serum as well as HBcAg expression level in the liver, which then determined the HBV persisternce, while the antiviral factors IFNγ, TNFα as well as immune negative regulatory factor PDL1 play important roles on HBV persistence.

## Background

Hepatitis B virus (HBV) infection is one of the major threats to public health worldwide and more than 240 million people are currently infected. Approximately 25% of the HBV infected individuals develop HBV-associated diseases afterwards, including liver failure, cirrhosis and hepatocellular carcinoma (HCC) [16].

An immunologically defined and reproducible small animal model for HBV infection remains unavailable due to the strict host specificity of HBV infection, which greatly hampers HBV related research. The laboratory mouse is genetically and immunologically well defined, and a large genetically modified animals are available for scientific research. However, mice could not be infected with HBV. Several lines of transgenic mice with replication competent HBV genomes have been established and showed powerful application value for HBV related research [6]. Nevertheless, transgenic mice have integrated HBV genome and HBV replication existed in all the hepatocytes. The presence of HBV genomes in these mouse lines inevitably induces host immune tolerance against HBV antigens, which is different from that occurs during a natural infection [1, 6, 9, 15]. In addition, the capability of production of HBV transgenic mouse line is not easy in ordinary laboratory conditions. Moreover, human liver transplanted mouse models were established and used for different studies [3, 5, 8]. However, The transplant models are based on immunodeficient mouse strains and difficult to operate in majority of laboratory.

Hydrodynamic injection (HI) of replication-competent HBV clone into the tail veins of mice can establish HBV replication in the liver of mouse [7, 18]. In 40% of C57/BL6 mice injected with 10 μg pAAV-HBV1.2 plasmid DNA, the persistence of HBV surface antigenemia (HBsAg) was more than 6 months. The tolerance against HBsAg in this model was due to the insufficient cellular immunity against HBV core antigen, as has been documented in humans [7]. The HBV HI mouse model is a highly interesting model for testing vaccination strategies and the mechanisms of viral persistence [4, 10, 20–22]. This model also could be used to evaluate replication competence of HBV constructs [10] as well as HBV related antiviral research [17]. Therefore, increasing the percentage of HBV persistent mice is very important to optimize the application of HBV HI mouse model.

In this study, we tested the impact of the dose of injected HBV plasmid DNA on HBV persistence in both C57/BL6 and BALB/c mice. In previous study, Huang et al. showed that there were 40% of the C57/BL6 mice injected with 10 μg pAAV-HBV1.2 plasmid DNA, serum HBsAg positive more than 6 months and none of the BALB/c mice injected with 10 μg pAAV-HBV1.2 plasmid DNA, serum HBsAg positive more than 4 weeks [7]. However, in our study, we found that 80% of the C57/BL6 mice receiving 5 μg pAAV-HBV1.2 plasmid DNA, serum HBsAg persisted more than 6 months. The HBV persistent rate was 2-fold increase compared with the results shown in previous study. Furthermore, we found that 60% of BALB/c mice receiving 5 μg of pAAV-HBV1.2 plasmid DNA, serum HBsAg persisted more than 3 months which showed a dramatic improvement compared with the results in previous study. On the contrary, C57/BL6 mice injected with 100 μg high dose of pAAV-HBV1.2 plasmid DNA had the highest HBsAg and HBeAg expression at the beginning after HI whereas rapid clearance of them afterwards. In addition, 1 μg low dose of pAAV-HBV1.2 plasmid DNA showed a slight impact on HBV persistence in BALB/c mice.

To find the possible mechanism of HBV persistence in this study, we further detected ALT levels in serum of the mice and the expression of interferon regulatory factor (IRF3), interferon stimulate genes (ISGs) including ISG15, 2′-5′-oligoadenylate synthase (OAS) and Proteinkinase R (PKR) as well as several immune factors including Interferon gamma (IFNγ), Tumor necrosis factor α (TNFα), Transforming growth factor β (TGFβ), Interleukin 6 (IL-6), IL-10, Programmed cell death ligand 1 (PDL1) in liver of the mice at 3 dpi. The elevated serum ALT levels were detected in all groups of HBV plasmid DNA injected mice at first day after HI, which was due to the liver damage caused by HI method. Furthermore, no significant difference in the mRNA levels of IRF3 and ISGs was detectable between the PBS injected control group and different concentrations of HBV plasmid DNA injected groups. However, we found that the antiviral factor IFNγ was significant up-regulated in liver of the mice injected with 1 μg or 100 μg pAAV-HBV1.2 plasmid DNA. TNFα was up-regulated significantly in liver of the mice injected with 100 μg pAAV-HBV1.2 plasmid DNA. Those results suggest that IFNγ and TNFα still play important roles in controlling virus infection in this study. Moreover, we found that the immune negative regulatory factor PDL1 was up-regulated significantly in liver of the mice injected with 5 μg pAAV-HBV1.2 plasmid DNA.

In conclusion, this study illustrated that HI with 5 μg medium dose of pAAV-HBV1.2 plasmid DNA significant increased the rates of HBV persistence in both C57/BL6 and BALB/c HI mouse model. The IRF3 and ISGs do not account for HBV clearance in our study. However, the antiviral factors IFNγ, TNFα as well as immune negative regulatory factor PDL1 play important roles on HBV persistence. In sum, this study will help us to further understand the mechanism of HBV persistence and better apply this model to explore new treatments against chronic HBV infection.

## Methods

### HI experiments

The pAAV-HBV1.2 plasmid contains an 1.2 fold over-length HBV genotype A genome (nt 1400-3182-1987) [7], was kindly provided by Prof. Pei-Jer Chen. The persistence of HBV in vivo was tested by HI method with different concentrations of HBV plasmid DNA in both C57/BL6 mice (male, 6–8 weeks old, from the breeding colonies of the experimental animal center in Shanghai, China) and BALB/c (male, 6–8 weeks old, from the breeding colonies of the experimental animal center in Hubei Province, China). HI experiments were carried out as described previously [7], 5 μg, 10 μg or 100 μg pAAV-HBV1.2 plasmid DNA were injected into the tail veins of C57/BL6 mice, separately and 1 μg, 5 μg or 10 μg pAAV-HBV1.2 plasmid DNA were injected into the tail veins of BALB/c mice, respectively, with 30 mice per group. Furthermore, to find the possible mechanism of the HBV persistence, 1 μg, 5 μg, 10 μg, or 100 μg pAAV-HBV1.2 plasmid DNA were injected into the tail veins of BALB/c mice, respectively, with five mice per group. The pAAV-HBV1.2 plasmid DNA were in a volume of 0.9% NaCl, equivalent to 8% of the mouse body weight. The total volume was delivered within 5–8 s. The control mice were injected only with the same volume of 0.9% NaCl, equivalent to 8% of the mouse body weight.

The animal experiments were carried out in concordance with the guidelines established by the Institutional Animal Care and Use Committee at the Huazhong University of Science and Technique and the Tongji Hospital of Tongji Medical College. The mice used in this study were anesthetized with ketamine and xylazine.

### Detection of ALT, HBsAg, HBeAg, HBcAb and HBsAb in mouse sera

Mouse sera from both C57/BL6 and BALB/c were collected at the 1d, 3d, 7d, 10d, 2w, 3w, 4w, 5w, 6w, 7w, 8w, 9w, 10w… after being injected with different concentrations of pAAV-HBV1.2 plasmid DNA. Alanine aminotransferase (ALT) levels of the BALB/c mice injected with 1 μg, 5 μg, 10 μg, or 100 μg pAAV-HBV1.2 plasmid DNA were measured on full automated biochemistry analyzer (7600 Series; Hitachi, Tokyo, Japan) by using ALT reagent (Denka Seiken, Tokyo, Japan).

The HBsAg, HBeAg, HBcAb and HBsAb were determined using commercial enzyme-linked immunosorbent assay (ELISA) kits (Kehua, Shanghai, China). Ten-fold diluted serum samples were used for detection.

### Isolation and analysis of HBV DNA in mouse sera

HBV DNA was extracted from 40 μl of mouse serum samples. The protocol was described previously [17], 40 μl of samples were treated with 10 μg DNase I for 16 h at 37 °C. 100 μl of lysis buffer (20 mM Tris-HCl, 20 mM EDTA, 50 mM NaCl and 0.5% SDS) containing 50 μg proteinase K were added. After incubation at 65 °C for 3 h, viral DNA was isolated by phenol/chloroform extraction and ethanol precipitation. The DNA pellet was rinsed with 70% ethanol and resuspended in 10 μl of ddH_2_O.

The quantification of HBV DNA was performed by using a routine real time PCR (qPCR), described previously [11–14, 19, 23]. The HBV copy numbers were determined by SYBR Green Real time PCR Master Mix (commercially available assay kit, TOYOBO, Osaka, Japan). Melt curve analysis and agarose gel electrophoresis were used to verify the specificity of the qPCR. The following primers were used: forward primer: 5′-CTG CAT CCT GCT GCT ATG-3′ (nt 408-425), reverse primer: 5′-CAC TGA ACA AAT GGC AC-3′ (nt 685-701) according to the reference sequence with Genbank accession number (AY220698.1). Serum containing a known concentration of HBV DNA was used as a positive control.

### Immunohistochemistry

The liver tissue was taken from the mice injected with different concentrations of pAAV-HBV1.2 plasmid DNA at 1, 3, 5, 9, 12 and 24 weeks post injection (wpi), and used for immunohistochemical staining of the HBcAg in hepatocytes. The liver tissue of the mice received 0.9% NaCl was used as negative control. Liver tissue was collected from the mice and embedded in paraffin. Intrahepatic HBcAg was visualized by immunohistochemical staining with rabbit anti-HBc (Dako) of the liver tissue sections. The liver tissue sections were also stained with hematoxylin.

### Purification of RNA from mouse liver tissue and real-time PCR detection

The BALB/c mice were killed at 3 day after being injected with 1 μg, 5 μg, 10 μg, or 100 μg pAAV-HBV1.2 plasmid DNA, respectively and receiving perfusion via the Hepatic portal vein. Total RNA was isolated from the liver tissue at 3 day post injection (dpi) by tissue RNA extraction kit (OMEGA, Norcross, USA) and used for analyzing the copy number of mouse IRF3, ISG15, OAS, PKR, IFNγ, TNFα, TGFβ, IL-6, IL-10 and PDL1 mRNA by using SYBR Green one-Step reverse trscription quantitive PCR (qRT-PCR) Kit (Takara, Dalian, China). Primers for qRT-PCR detection were provided by Qiagen Company (Qiagen, Hilden, Germany). β-actin was used as housekeeping gene to normalize qRT-PCR results.

### Statistical analysis

The data were evaluated by Student’s t-test or one-way ANOVA followed by Tukey’s post hoc test. The statistical significance of the data was considered at *p* < 0.05.

## Results

### Kinetics of HBsAg, HBeAg, HBcAb, HBsAb in sera of both C57/BL6 and BALB/c mice after HI

**Fig. 1:**
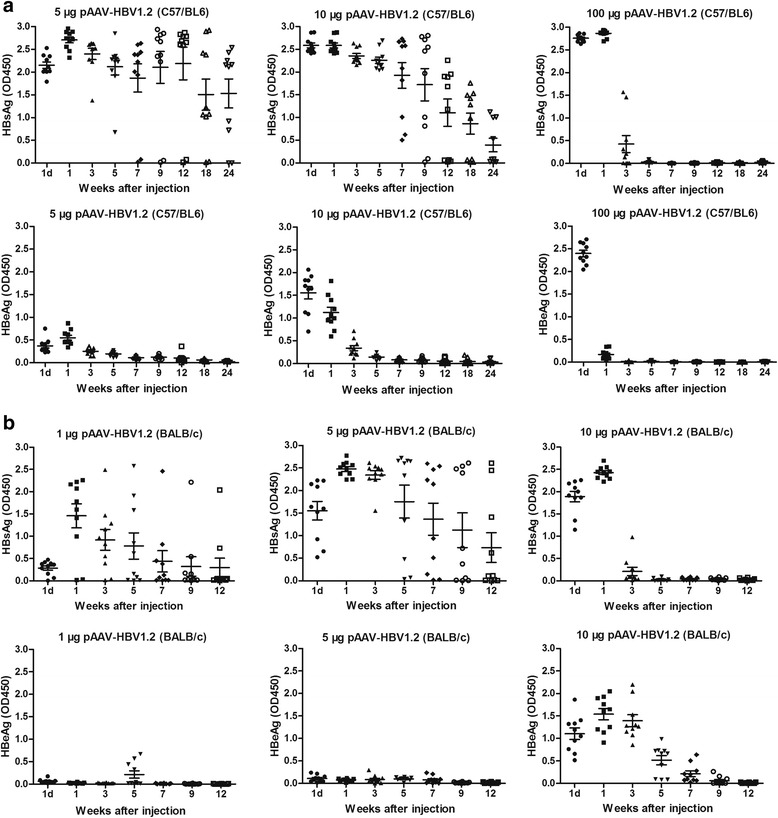
Kinetics of HBsAg and HBeAg expression level in mice treated with different concentrations of HBV plasmid DNA. C57/BL6 mice HI with 5 μg, 10 μg or 100 μg pAAV-HBV1.2 plasmid DNA and BALB/c mice HI with 1 μg, 5 μg or 10 μg pAAV-HBV1.2 plasmid DNA, respectively. The sera of mice were collected at different time points after HI. At least 10 mice per group were analyzed. HBsAg and HBeAg levels in the sera of **a** C57/BL6 or **b** BALB/c mice after HI with different dose of HBV plasmid DNA

To investigate whether the dose of injected HBV plasmids has a great impact on HBV persistence, 5 μg, 10 μg or 100 μg pAAV-HBV1.2 plasmid DNA were injected into the C57/BL6 mice, respectively by HI method. Figure 1a showed the HBsAg and HBeAg levels in serum of the C57/BL6 mice. We can see that in the group injected with 5 μg pAAV-HBV1.2 plasmid DNA, serum HBsAg peaked at 1 wpi. Most of the mice were still showed HBsAg positive at 24 wpi. In the group injected with 10 μg pAAV-HBV1.2 plasmid DNA, HBsAg was peaked at 1 dpi. Half of the mice were still showed HBsAg positive at 24 wpi. However, in the group injected with 100 μg pAAV-HBV1.2 plasmid DNA, HBsAg was peaked at 1 dpi and were cleared completely after 5 wpi. Moreover, in the group injected with 5 μg pAAV-HBV1.2 plasmid DNA, the HBeAg was also peaked at 1 wpi, but kept at a very low level during the whole process. However, in the groups injected with 10 μg or 100 μg pAAV-HBV1.2 plasmid DNA, HBeAg levels were peaked at 1 dpi. In addition, at first day after HI, the average value of HBeAg in the 100 μg pAAV-HBV1.2 plasmid DNA injected group was the highest one among those three groups whereas dropped very quickly afterwards, and the HBeAg of all the mice in this group were not detectable after 3 wpi.

To know whether the concentrations of pAAV-HBV1.2 plasmid DNA less than 5 μg could induce higher rate of HBV persistence, 1 μg, 5 μg or 10 μg pAAV-HBV1.2 plasmid DNA were injected into the BALB/c mice, respectively by HI method. Figure 1b showed the HBsAg and HBeAg levels in serum of the BALB/c mice. The HBsAg in all of the three groups were peaked at 1 wpi. However, there were more HBsAg postive mice in 5 μg pAAV-HBV1.2 plasmid DNA injected group than the other two groups at 12 wpi. HBeAg were peaked at 5 weeks or 1 week after the mice being injected with 1 μg or 10 μg of pAAV-HBV1.2 plasmid DNA, respectively. Furthermore, we did not see obvious peak of HBeAg in serum of the 5 μg pAAV-HBV1.2 plasmid DNA injected mice during the whole process. The kinetics of HBsAg and HBeAg in serum of the mice illustrated that the onset dose of HBV plasmid DNA has a great impact on the HBV persistence in both C57/BL6 and BALB/c HI mouse model.

In addition, we found that all of the C57/BL6 mice injected with 5 μg pAAV-HBV1.2 plasmid DNA failed to develop neutralizing anti-HBs and most of BALB/c mice injected with 5 μg pAAV-HBV1.2 plasmid DNA could not produce HBs antibodies (Table 1), indicating a tolerance status toward HBsAg in vivo.

**Table 1:** Appearance of anti-HBc and anti-HBs antibodies in C57/BL6 and BALB/c mice after HI with different concentrations of HBV plasmid DNA

	Anti-HBc (C57/BL6)		Anti-HBs (C57/BL6)		
HBV DNA	Day 4	Day 7	Day 10	Day 20	Day 30
5 μg pAAV-HBV1.2	3/10	10/10	0/10	0/10	0/10
10 μg pAAV-HBV1.2	6/10	10/10	0/10	0/10	2/10
100 μg pAAV-HBV1.2	9/10	10/10	0/10	4/10	8/10
	Anti-HBc (BALB/c)		Anti-HBs (BALB/c)		
HBV DNA	Day 4	Day 7	Day 10	Day 20	Day 30
1 μg pAAV-HBV1.2	0/10	10/10	0/10	2/10	4/10
5 μg pAAV-HBV1.2	4/10	10/10	0/10	0/10	2/10
10 μg pAAV-HBV1.2	7/10	10/10	0/10	6/10	10/10

C57/BL6 mice HI with 5 μg, 10 μg or 100 μg pAAV-HBV1.2 plasmid DNA and BALB/c mice injected with 1 μg, 5 μg or 10 μg pAAV-HBV1.2 plasmid DNA separately. The number of animals in each group positive for anti-HBc or anti-HBs antibodies

### Expression of HBcAg in liver of the C57/BL6 and BALB/c mice

Immunohistochemical staining was performed to determine the expression of HBcAg in the liver sections of the mice at 1, 3, 5, 9 and 12 wpi.

**Fig. 2:**
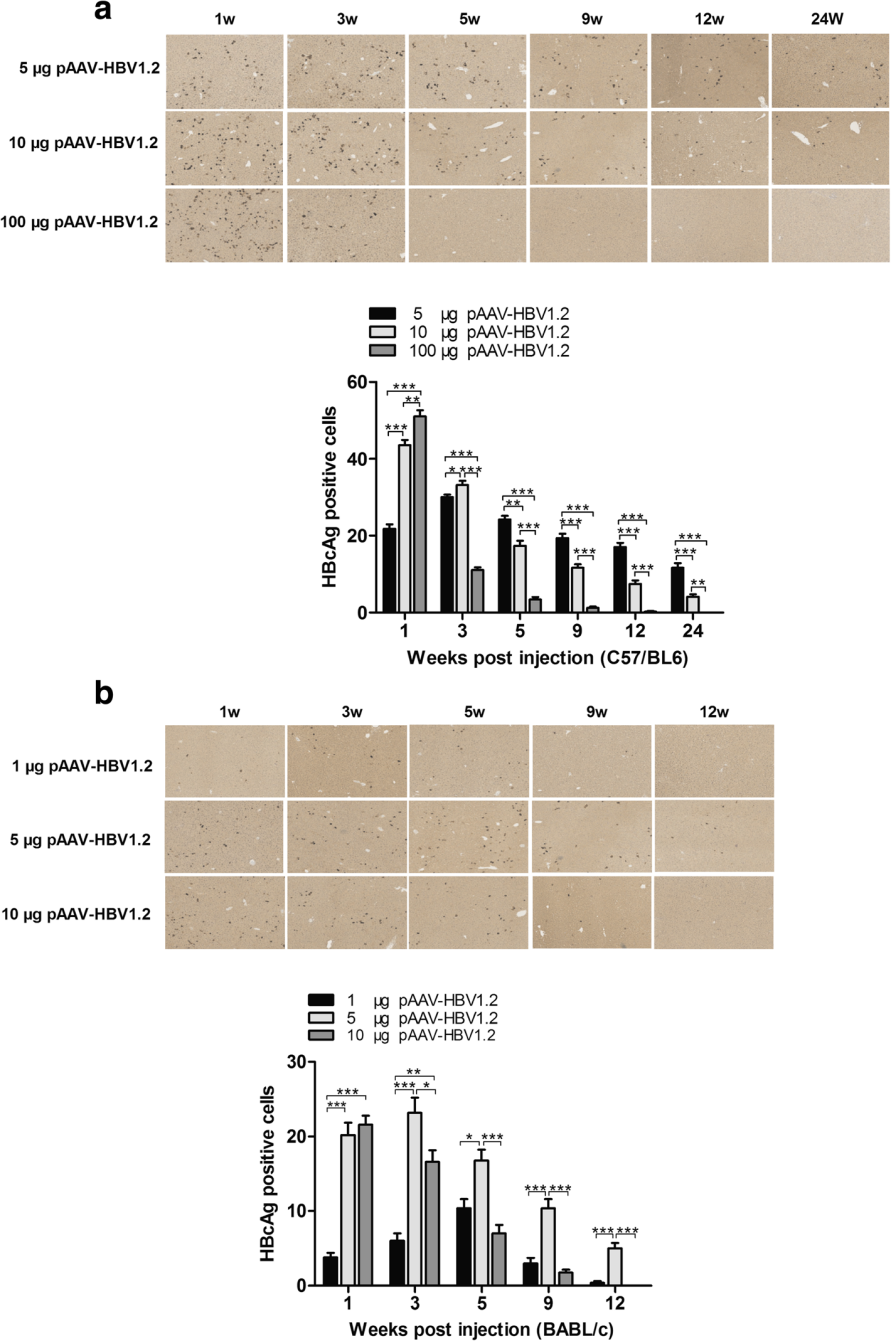
HBcAg expression in liver of the C57/BL6 and BALB/c mice after HI with different concentrations of HBV plasmid DNA. 5 μg, 10 μg or 100 μg pAAV-HBV1.2 plasmid DNA were injected into C57/BL6 mice and 1 μg and 5 μg or 10 μg pAAV-HBV1.2 plasmid DNA were injected into BALB/c mice by HI method, separately. At 1, 3, 5, 9, 12 and 24 wpi, mice were sacrificed and livers were analyzed. Immunohistochemical staining of the liver sections for HBcAg in hepatocytes and frequencies of HBcAg positive cells of **a** C57/BL6 or **b** BALB/c mice. At least three mice per group were analyzed. The data were analyzed by One-way ANOVA. Statistically significant differences between the different groups were indicated (*means *p* < 0.05, **means *p* < 0.01 and ***means *p* < 0.001)

The results demonstrated that HBcAg positive cells could be detected in the liver sections of both C57/BL6 and BALB/c mice after being injected with each different concentrations of the HBV plasmid DNA. Figure 2a showed the HBcAg expression in liver of the C57/BL6 mice. The number of HBcAg positive cells in liver of the 10 μg or 100 μg pAAV-HBV1.2 plasmid DNA injected mice were peaked at 1 wpi and most abundant in liver of the 100 μg pAAV-HBV1.2 plasmid DNA injected mice. However, the number of HBcAg positive cells in liver of the 5 μg pAAV-HBV1.2 plasmid DNA injected mice was peaked at 3 wpi. Figure 2b showed the HBcAg expression in liver of the BALB/c mice. The HBcAg positive cells peaked at 1 week after the mice being injected with 10 μg pAAV-HBV1.2 plasmid DNA whereas the HBcAg positive cells were peaked at 5 weeks or 3 weeks after the mice being injected with 1 μg pAAV-HBV1.2 plasmid DNA or 5 μg pAAV-HBV1.2 plasmid DNA, separately.

These data indicated that the number of the HBcAg positive cells in liver of the higher dose of pAAV-HBV1.2 plasmid DNA injected mice peaked earlier than those of the lower dose of pAAV-HBV1.2 plasmid DNA injected mice.

### HBV DNA levels in C57/BL6 and BALB/c mice after HI

**Fig. 3:**
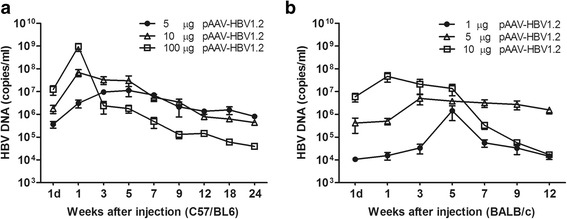
HBV DNA levels in the sera of C57/BL6 and BALB/c mice after HI with different dose of HBV plasmid DNA. 5 μg, 10 μg or 100 μg pAAV-HBV1.2 plasmid DNA were injected into C57/BL6 mice and 1 μg and 5 μg or 10 μg pAAV-HBV1.2 plasmid DNA were injected into BALB/c mice separately. HBV DNA were extracted from the sera of the mice which collected at indicated time points and detected by qRT-PCR analyses. At least four mice per group were analyzed. HBV DNA copies in the sera of **a** C57/BL6 or **b** BALB/c mice after HI

To further investigate the relationship between HBV replication competence and the concentrations of injected pAAV-HBV1.2 plasmid DNA in vivo. Mouse serum samples were collected for HBV DNA quantification at indicated time points. Figure 3a showed the HBV DNA levels in the C57/BL6 mice. HBV DNA levels of all the C57/BL6 mice were peaked at 1 wpi. The copy number of HBV DNA in the serum of 100 μg pAAV-HBV1.2 plasmid DNA injected mice was around 10^9^/ml at 1 wpi, which was much higher than that of 10 μg pAAV-HBV1.2 (~10^8^/ml) or 5 μg pAAV-HBV1.2 (~5 × 10^6^/ml) plasmid DNA injected mice. However, they droped very quickly and maintained at lower levels after 3 wpi. Figure 3b demonstrated the HBV DNA levels in the BALB/c mice. We can see that the HBV DNA levels peaked at 3 weeks after the mice being injected with 5 μg pAAV-HBV1.2 plasmid DNA. However, the HBV DNA levels peaked at 5 weeks or 1 week after the mice being injected with 1 μg or 10 μg pAAV-HBV1.2 plasmid DNA, respectively. The results showed that the tendency of HBV DNA levels were almost consistent with the HBsAg levels in serum of the mice.

### Comparison of HBsAg persistence in C57/BL6 and BALB/c mice after HI

**Fig. 4:**
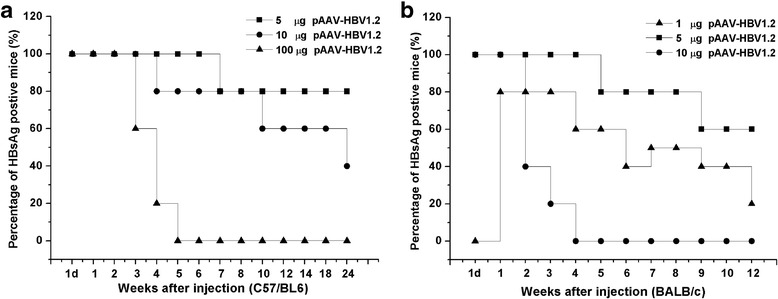
HBV persistence in both C57/BL6 and BALB/c mouse after HI with different concentrations of HBV plasmid DNA. 5 μg, 10 μg or 100 μg pAAV-HBV1.2 plasmid DNA were injected into C57/BL6 mice and 1 μg and 5 μg or 10 μg pAAV-HBV1.2 plasmid DNA were injected into BALB/c mice, separately. The sera of mice were collected at different time points after HI. At least 10 mice per group were analyzed. The positive rate for serum HBsAg in **a** C57/BL6 or **b** BALB/c mice receiving different concentrations of pAAV-HBV1.2 plasmid DNA

We monitored the dynamic variation of the serum HBsAg in both C57/BL6 and BALB/c mice after receiving different dose of pAAV-HBV1.2 plasmid DNA. Figure 4a showed that 80% of the C57/BL6 mice receiving 5 μg pAAV-HBV1.2 plasmid DNA, serum HBsAg persistent more than 6 months. The percentage of HBsAg positive mice was double of the 10 μg pAAV-HBV1.2 plasmid DNA injected mice. Figure 4b showed that 60% of the BALB/c mice injected with 5 μg pAAV-HBV1.2 plasmid DNA, serum HBsAg persistent more than 3 months after HI. As we know, in previous study, Huang et al. [7] showed that only 40% of the C57/BL6 mice injected with 10 μg pAAV-HBV1.2 plasmid DNA showed serum HBsAg persistent more than 6 months, whereas none of the 10 μg pAAV-HBV1.2 plasmid DNA injected BALB/c mice demonstrated serum HBsAg positive over 4 weeks. Nevertheless, our results indicated that 5 μg pAAV-HBV1.2 plasmid DNA could dramatically increase HBV persistent rates in both C57/BL6 and BALB/c mice.

### ALT levels in serum and the mRNA levels of IRF3, ISGs and immune factors in liver of the BALB/c mice after HI

**Fig. 5:**
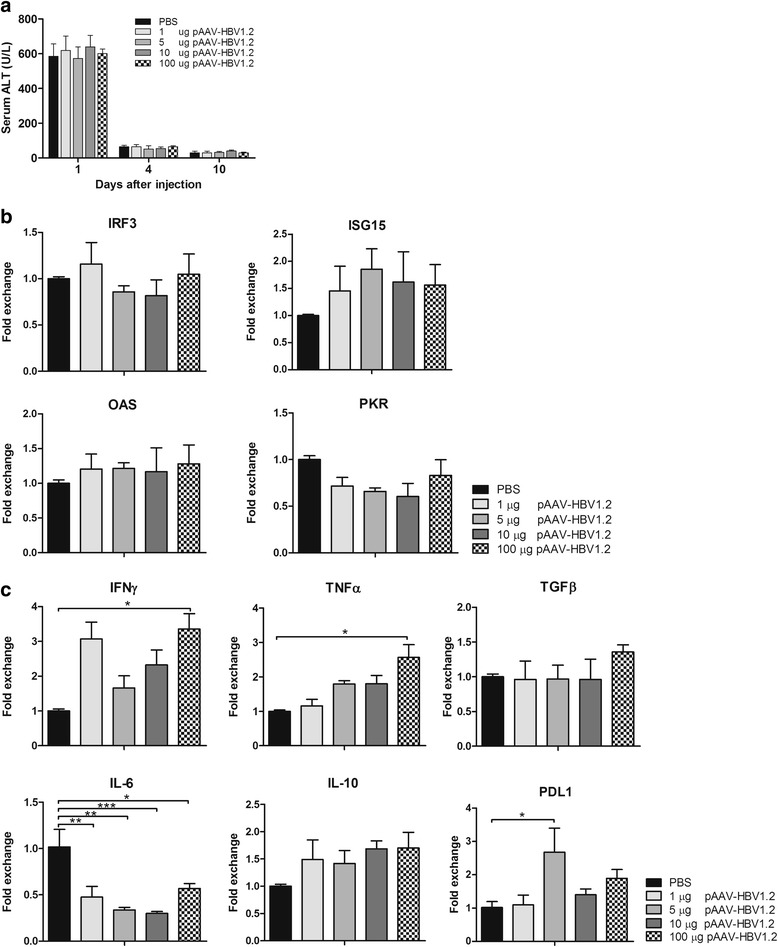
ALT and mRNA levels of ISGs and immune factors after HI with different concentrations of HBV plasmid DNA. 1 μg, 5 μg, 10 μg or 100 μg pAAV-HBV1.2 plasmid DNA were injected into BALB/c mice, respectively. The mice sera were collected at 1, 4, 10 dpi and the total mRNA was extracted form liver tissue of the mice at 3 dpi. **a** Serum ALT levels were measured on full automated biochemistry analyzer and the mRNA expression levels of **b** IRF3, ISG15, OAS, PKR and **c** IFNγ, TNFα, TGFβ, IL-6, IL-10 and PDL1 were determined by qRT-PCR. β-actin was used for normalization. Each sample was run in duplicate and at least four mice per group were analyzed. Differences between the groups were analyzed by using the One-way ANOVA. Statistically significant differences between the pAAV-HBV1.2 plasmid DNA injected groups and the PBS injected control group were indicated by * for *p* < 0.05, ** for *p* < 0.01 and *** for *p* < 0.001

To find a possible mechanism correlated with HBV persistence in vivo, we analyzed the ALT levels in serum and mRNA levels of IRF3 and ISGs including ISG15, OAS, PKR in liver of the mice. Figure 5a showed the ALT levels in serum of the mice. The elevated serum ALT levels were detected in the mice of all the groups at first day after HI. The elevated ALT levels were due to liver damage caused by HI method. Figure 5b showed the mRNA levels of IRF3, ISG15, OAS and PKR in liver of the BALB/c mice injected with 1 μg, 5 μg, 10 μg, or 100 μg pAAV-HBV1.2 plasmid DNA. No significant difference in the mRNA levels of IRF3 and ISGs was detectable between the PBS injected control group and different concentrations of pAAV-HBV1.2 plasmid DNA injected groups, indicating that the antiviral ISGs were not account for HBV clearance in our study.

To further exploit the potential mechanism of HBV persistence in our study, the mRNA levels of cytokines including IFNγ, TNFα, TGFβ, IL-6, IL-10 and PDL1 in liver of the BALB/c were tested at 3 day after the mice being injected with 1 μg, 5 μg, 10 μg, or 100 μg pAAV-HBV1.2 plasmid DNA. As shown in Fig. 5c, antiviral factor IFNγ was significantly up-regulated in liver of the mice injected with 1 μg or 100 μg pAAV-HBV1.2 plasmid DNA. TNFα was up-regulated significantly in liver of the mice injected with 100 μg pAAV-HBV1.2 plasmid DNA. The results indicate that IFNγ and TNFα still play important roles to control virus infection in this study. Moreover, pro-inflammatory factor IL-6 was down-regulated and anti-inflammatory factor IL-10 and TGFβ were slightly up-regulated in all of the pAAV-HBV1.2 plasmid DNA injected BALB/c mice, suggesting that the virus could inhibit host immune response to resist host defence. In addition, the immune negative regulatory factor PDL1 was up-regulated significantly in liver of the 5 μg pAAV-HBV1.2 plasmid DNA injected mice. Those results illustrated that IFNγ, TNFα as well as immune negative regulatory factor PDL1 play important roles on HBV persistence.

## Discussion

Establishment of a stable as well as genetically and immunologically defined mouse model is very important to accelerate HBV related basic and clinical research process. HBV HI mouse model is a HBV replication-competent mouse model which showed usual definition of persistent HBV infection in humans. It is a very valuable model that will help to further understand the mechanism of HBV tolerance and also provide a useful tool for HBV related antiviral research. In previous study, they found that both vector construction and mouse genetic background determined HBV persistence in this model. The tolerance toward HBV surface antigen in HI mouse model was shown to be due to an insufficient cellular immunity against HBcAg, as was documented in humans [7].

As we know, HBV persistent mouse model is needed for HBV related antiviral research. However, the previous study showed that only 40% of the C57/BL6 mice injected with 10 μg pAAV-HBV1.2 plasmid DNA, serum HBsAg positive more than 6 months and none of the BALB/c mice injected with 10 μg pAAV-HBV1.2 plasmid DNA, serum HBsAg positive more than 4 weeks [7]. Therefore, optimization of the existing HBV HI mouse model is necessary to accelerate the widespread application of this model. The previous study showed that the size of the viral inoculum contributes to the outcome of HBV infection in adult chimpanzees. They found that CD4^+^ T-cell depletion before inoculation of a normally rapidly controlled inoculum precluded T-cell priming and caused persistent infection with minimal immunopathology, suggesting that the relationship between the kinetics of viral spread and CD4^+^ T-cell priming determines the outcome of HBV infection [2]. However, it should be noted that Asabe et al. used adult chimpanzees in their study so that the age, sex and genetic diversity of the chimpanzees probably have an impact on the outcome of HBV infection. However, in our study, 5 weeks old genetically and immunologically defined laboratory mice were used so that the dose of the HBV plasmid DNA should be the unique factor which account for the outcome of HBV invasion.

Therefore, to test whether the dose of the HBV plasmid DNA influence the course and outcome of HBV infection in HI mouse model, we start with injection of different concentrations of pAAV-HBV1.2 plasmid DNA in the C57/BL6 mice. The results demonstrated that there were 80% of the C57/BL6 mice showing HBsAg positive more than 6 months after being injected with 5 μg pAAV-HBV1.2 plasmid DNA. It was two fold increase compared with the mice injected with 10 μg pAAV-HBV1.2 plasmid DNA in previous study. Whereas, the viruses in the mice injected with 100 μg higher dose of pAAV-HBV1.2 plasmid DNA were cleared quickly with declined viral kinetics even if high HBsAg and HBeAg levels appeared at the first day after HI. To further confirm the results and investigate whether the lower inocula could promote HBV persistence, BALB/c mice were injected with 1 μg, 5 μg or 10 μg pAAV-HBV1.2 plasmid DNA, separately. We found that there were 60% of the BALB/c mice showing HBsAg positive more than 3 months after being injected with 5 μg pAAV-HBV1.2 plasmid DNA. This was a significant improvement compared with the previous results which illuminated that none of the BALB/c mice showing HBsAg positive more than 4 weeks after being injected with 10 μg pAAV-HBV1.2 plasmid DNA. In addition, we found that injection of 1 μg lower dose of pAAV-HBV1.2 plasmid DNA just exert a slight influence on the course and outcome of HBV invasion in this model. The inability to produce anti-HBs after injection of 5 μg pAAV-HBV1.2 plasmid DNA into both C57/BL6 and BALB/c mice (As shown in Table 1) suggesting that the tolerance against HBsAg was generated in vivo.

To investigate the possible mechanism of HBV persistence in this study, we detected the ALT levels in serum and the expression of IRF3, ISGs as well as several immune factors in liver of the mice at 3 day after the BALB/c mice being injected with 1 μg, 5 μg, 10 μg, or 100 μg pAAV-HBV1.2 plasmid DNA. We found that the elevated serum ALT levels in the mice of all the groups at first day after HI were due to the liver damage caused by HI method. Furthermore, no significant difference in the mRNA levels of IRF3 and ISGs was detectable between the PBS injected control group and different concentrations of pAAV-HBV1.2 plasmid DNA injected groups. However, we found that the antiviral factor IFNγ was significantly up-regulated in liver of the mice injected with 1 μg or 100 μg pAAV-HBV1.2 plasmid DNA. TNFα was up-regulated significantly in liver of the mice injected with 100 μg pAAV-HBV1.2 plasmid DNA. Moreover, the immune negative regulatory factor PDL1 was up-regulated significantly in liver of the 5 μg pAAV-HBV1.2 plasmid DNA injected mice. Taken together, the results highlight that the IRF3 and ISGs do not account for HBV clearance but IFNγ, TNFα as well as immune negative regulatory factor PDL1 play important roles on HBV persistence in HI mouse model.

In a word, our study demonstrated the significant increases of HBV persistent rates in both C57/BL6 and BALB/c HI mouse model after being injected with 5 μg pAAV-HBV1.2 plasmid DNA, which will greatly improve the application of this model. As we know, a lot of HBV related antiviral researches are based on HBV chronic infection mouse model. Therefore, increasing the percentage of HBsAg positive mice in HBV HI mouse model will greatly reduce the number of experimental animals. In addition, our study also provide an opportunity to better understand the mechanism of HBV persistence in HI mouse model, which is very important to disclose the host immune response against HBV invasion. Taking togethr, here we provide a valuable tool which will contribute a lot to the HBV related basic research and also useful for the evaluation of anti-HBV therapy.

## Conclusion

In this paper we demonstrated that, in the HBV HI mouse model, the different concentrations of the injected pAAV-HBV1.2 plasmid DNA contribute to the diverse kinetics of HBsAg and HBeAg levels in the serum as well as HBcAg expression in the liver, which then determined the HBV persistence. In addition, we found that the antiviral factors IFNγ, TNFα as well as immune negative regulatory factor PDL1 play important roles on HBV persistence. In brief, here we optimized the HBV HI mouse model which will greatly improve the application of this model for HBV related research.
